# Supplementary figures and images for: An expression and function analysis of the CXCR4/SDF-1 signalling axis during pituitary gland development
Source: PLoS One. 2023 Feb 17;18(2):e0280001. doi: 10.1371/journal.pone.0280001 (PMC9937476; doi:10.1371/journal.pone.0280001)

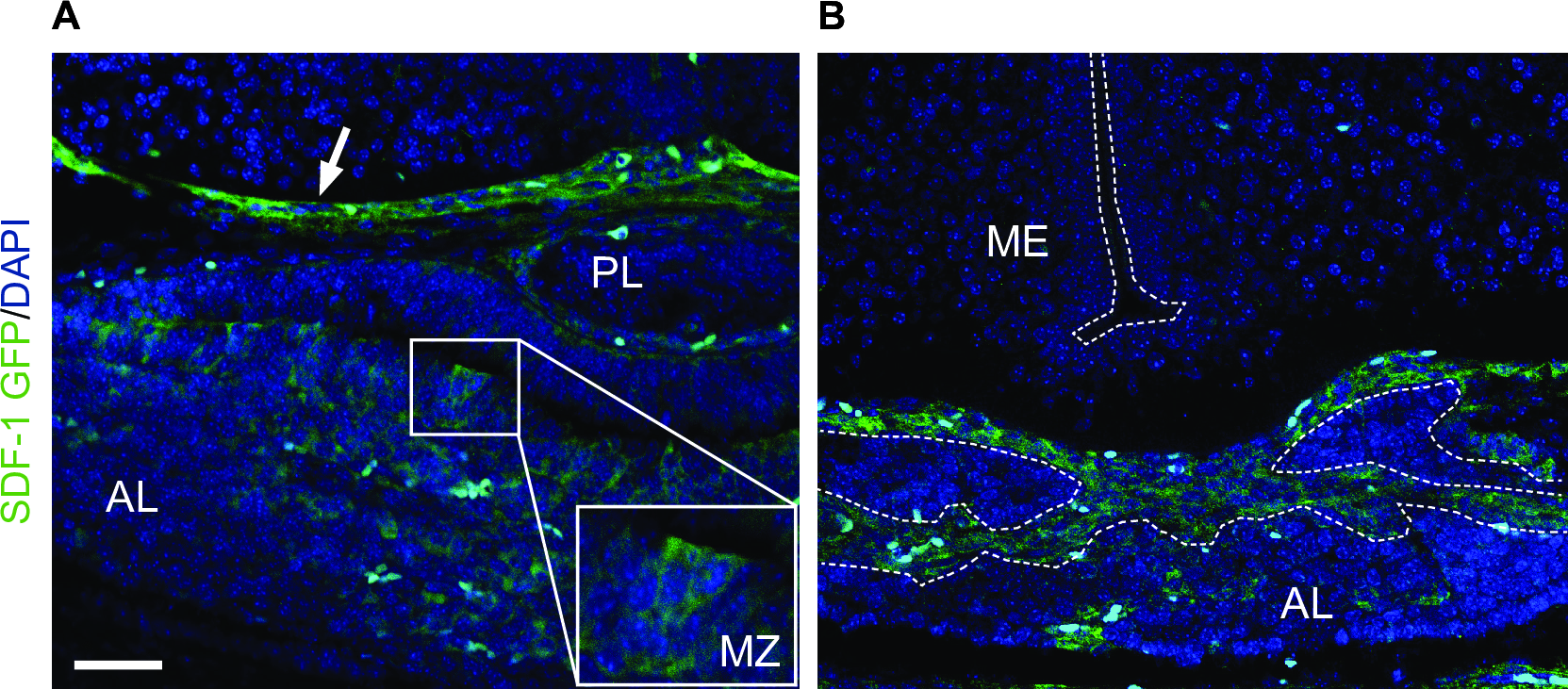

Supplement: S1 Fig — (A) SDF-1-GFP expression is observed in supportive mesenchyme surrounding the pituitary gland (arrow). SDF-1-GFP is expressed within the pituitary anterior lobe (AL), particularly in cells of the marginal zone (MZ) (inset). (B) Anterior-most region of the pituitary gland where SDF-1-GFP is expressed in stromal cells intermingling with the AL (delimited by dotted line). Counterstain is DAPI. Scale bar: 50 μm. (TIF) [file pone.0280001.s001.tif]

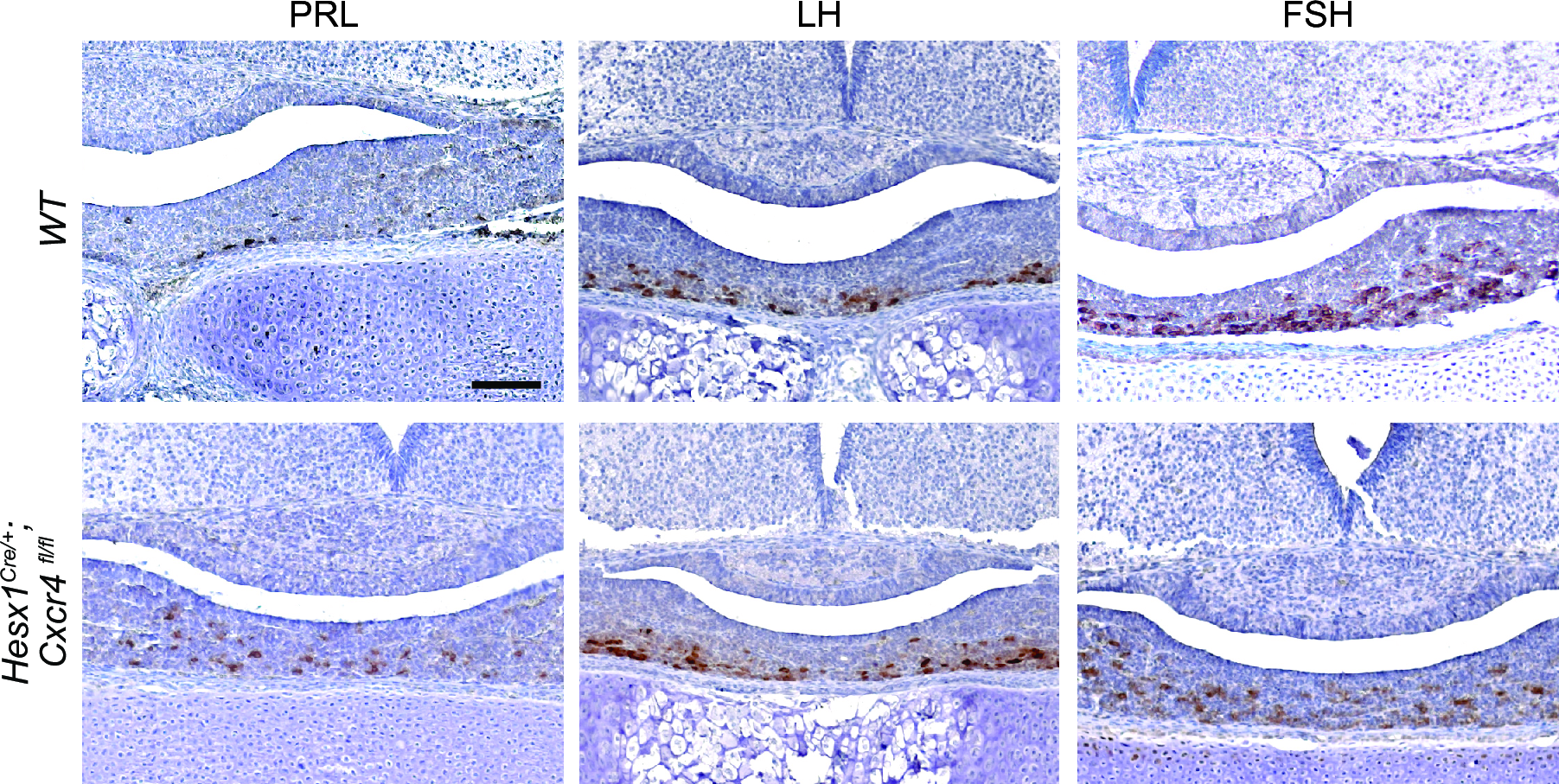

Supplement: S2 Fig — Staining in in wild type (top row) and Hesx1Cre/+;Cxcr4fl/fl (bottom row) pituitaries at 18.5 dpc shows normal differentiation of these endocrine populations. Counterstain is haematoxylin. Scale bar: 100 μm. (TIF) [file pone.0280001.s002.tif]

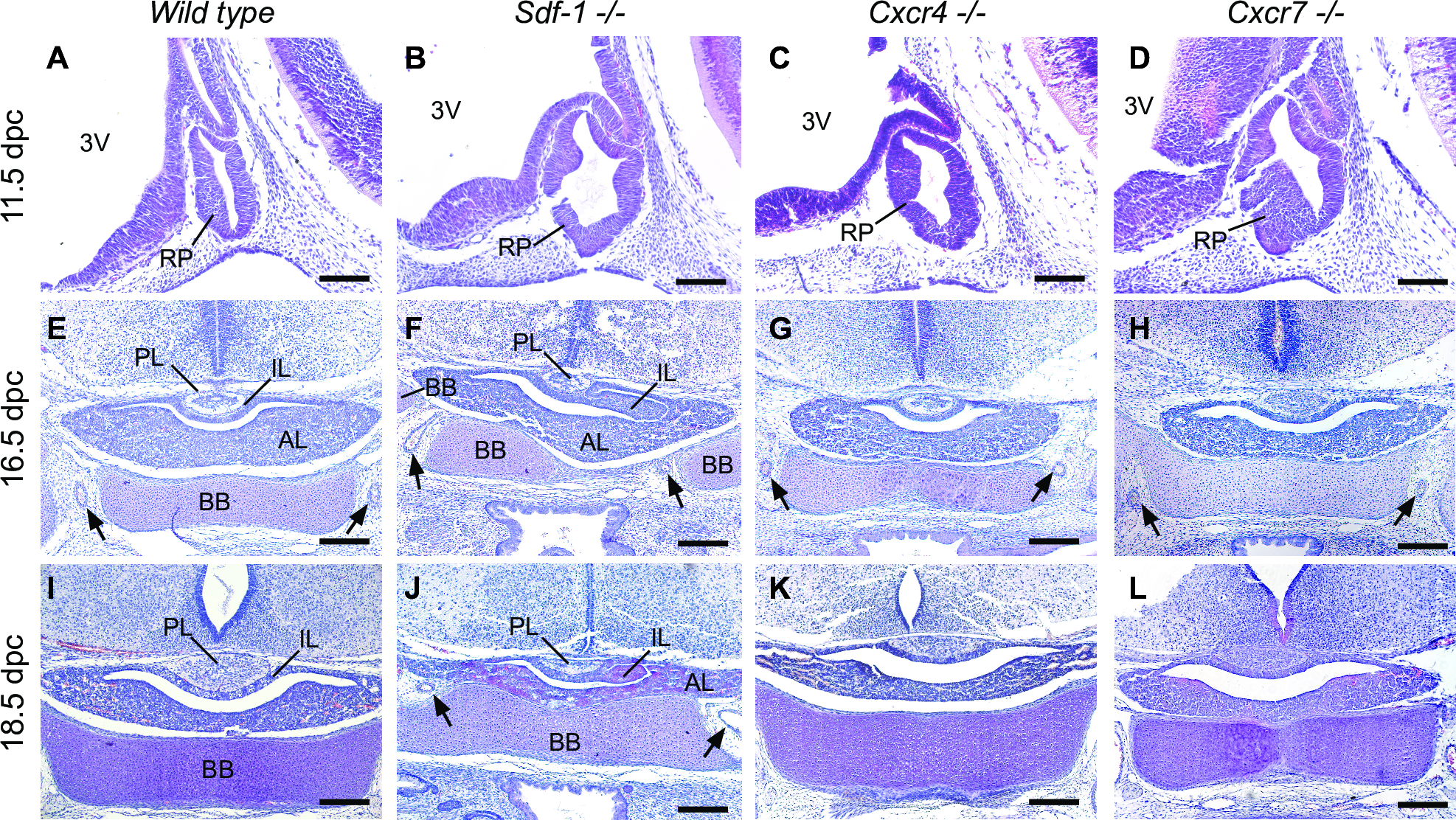

Supplement: S3 Fig — (A-D) At 11.5 dpc, the RP from Sdf-1-/-, Cxcr4-/- and Cxcr7-/- embryos is not affected. (E) At 16.5 dpc the wild type developing pituitary sits on top of the basisphenoid bone (BB), which is flanked by large blood vessels (arrows). (F) Sdf-1-/- pituitaries at this stage have an abnormal shape, invaginations of the intermediate lobe (IL) and a deformed BB. Note ectopic blood vessels intermingling in between the BB (arrows). (G-H) Cxcr4-/- and Cxcr7-/- pituitaries resemble wild types at 16.5 dpc. (I-J) The Sdf-1-/- pituitary phenotype at 18.5 dpc also presents an invaginated IL and deformed BB with ectopic blood vessels (arrows). (K-L) Cxcr4-/- and Cxcr7-/- pituitaries are morphologically normal at 18.5 dpc. Scale bars: A-D: 100 μm; E-L: 200 μm. RP: Rathke’s Pouch; 3V: Third Ventricle; PL: Posterior Lobe; AL: Anterior Lobe; IL: Intermediate Lobe; BB: Basisphenoid Bone. (TIF) [file pone.0280001.s003.tif]

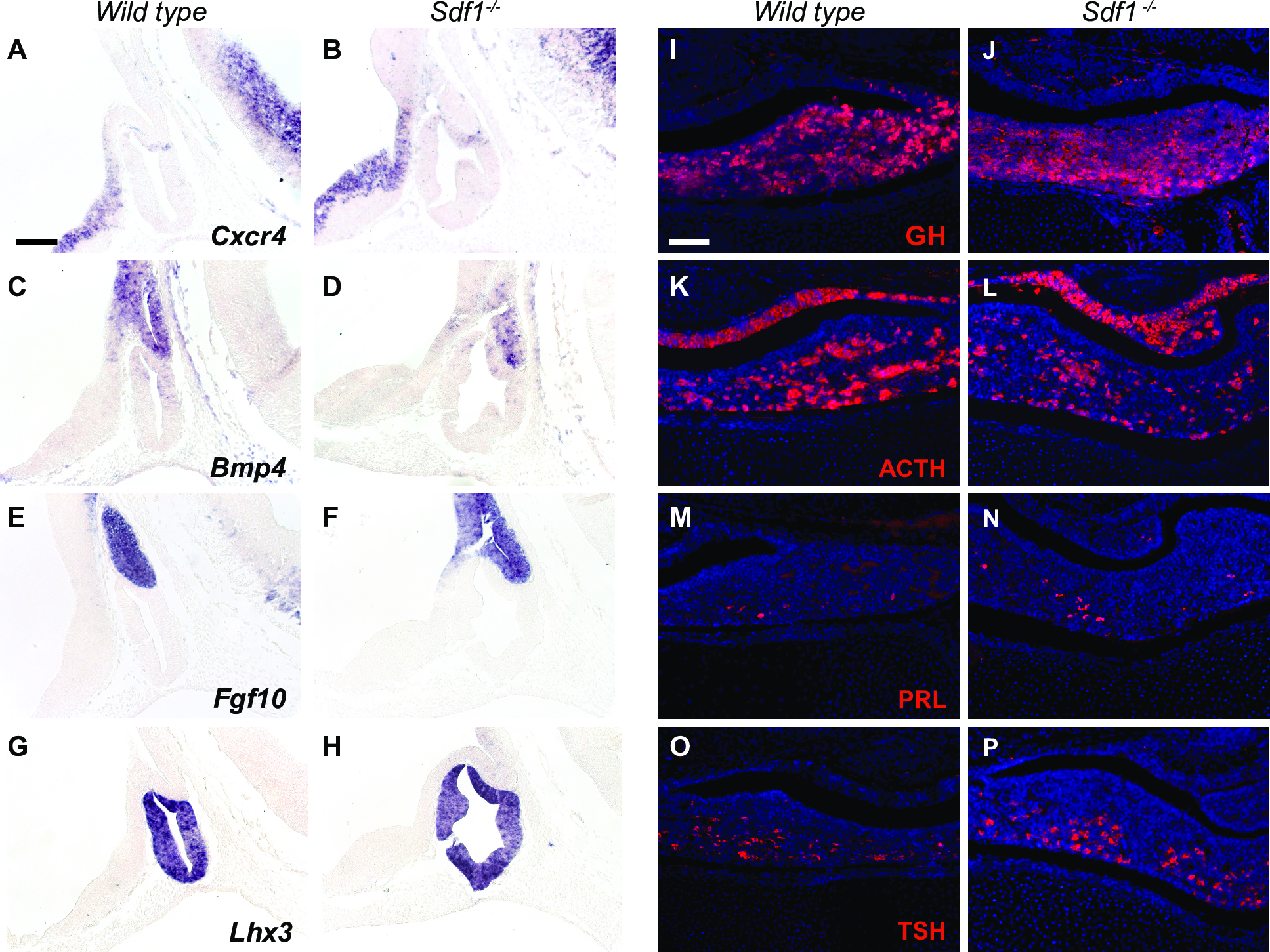

Supplement: S4 Fig — (A-B) In situ hybridisation in 11.5 dpc embryos shows that Cxcr4 expression is not significantly altered in Sdf-1-/- mutants. (C-F) No differences are found in the expression domains of hypothalamic factors Bmp4 and Fgf10. (G-H) The expression of the transcription factor Lhx3 (necessary for normal pituitary development) is not altered in Sdf-1-/- mutants. (I-P) Fluorescence immunostaining against different pituitary hormones at 18.5 dpc shows proper terminal differentiation of the pituitary endocrine lineages in Sdf-1-/- mutants. DAPI counterstain: K-T. Scale bars: 100 μm. GH: Growth Hormone; ACTH: Adeno-Corticotropic Hormone; TSH: Thyroid Stimulating Hormone. (TIF) [file pone.0280001.s004.tif]

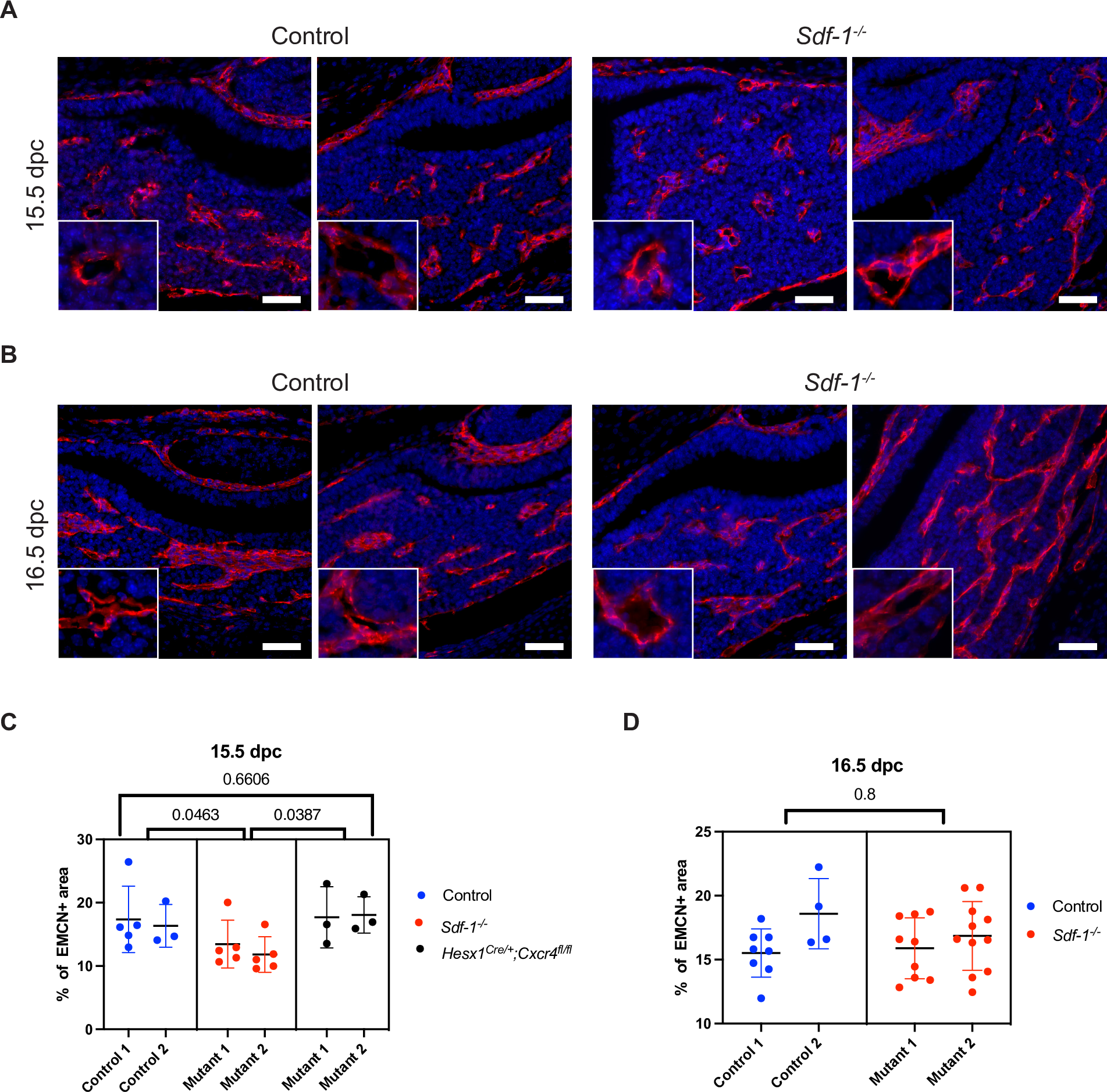

Supplement: S5 Fig — (A) Immunofluorescent stainings for the endothelial marker Endomucin (EMCN) in control (left panel) and Sdf-1-/- (right panel) pituitaries at 15.5 dpc. (B) EMCN stainings in 16.5 dpc control (left panel) and Sdf-1-/- (right panel) pituitaries. Representative images from two biological replicates are shown for each genotype. Insets in all panels show representative close-ups of EMCN+ cells present in luminal structures. DAPI counterstain. Scale bars: 50 μm (10 μm for insets). (C) Quantification of the percentage of area covered by EMCN+ cells in Sdf-1+/+, Sdf-1-/- and Hesx1Cre/+;Cxcr4fl/fl pituitary sections at 15.5 dpc (n = 2, nested one-way ANOVA with Holm-Šídák’s multiple comparisons test, P = 0.02). (D) Quantification of the percentage of area covered by EMCN+ cells in Sdf-1+/+ and Sdf-1-/- pituitary sections at 16.5 dpc (n = 2, nested t-test, P = 0.8). Each data point represents a different section. Horizontal black lines in plots represent mean values and error bars show standard deviations. Significance values are shown for each pair-wise comparison. (TIF) [file pone.0280001.s005.tif]
